# Supplementary material for: Preoperative Factors for Lymphovascular Invasion in Prostate Cancer: A Systematic Review and Meta-Analysis
Source: Int J Mol Sci. 2024 Jan 10;25(2):856. doi: 10.3390/ijms25020856 (PMC10815768; doi:10.3390/ijms25020856)
Supplement: Supplementary file 1 [file ijms-25-00856-s001.zip › ijms-2753607-supplementary.pdf]

Table S1. Quality assessment of studies included in the systematic review.

Study	Selection				Comparability	Exposure			Total quality scores
	**Adequacy of the case definition**	**Representativeness of the cases**	**Selection of controls**	**Definition of controls**	**Comparability of cases and controls on the basis of the design or analysis**	**Ascertainment of exposure**	**Same method of ascertainment for cases and controls**	**Non-response rate**	
Antunes et al.	★	★	★	★	★★	★	★	-	8
Brooks et al.	★	-	★	★	★★	★	★	-	7
Cheng et al.	★	★	★	★	★★	★	★	-	8
D'Andrea et al.	★	★	★	★	★	★	★	-	7
De La Taille et al.	★	★	★	★	★★	★	★	-	8
Elharram et al.	-	-	★	★	★	★	★	-	5
Fajkovic et al.	★	★	★	★	★★	★	★	-	8
Fawzy et al.	-	★	★	★	★	★	★	-	6
Ferrari et al.	★	★	★	★	★★	★	★	-	8
Galiabovitch et al.	★	★	★	★	★★	★	★	-	8
Gesztes et al.	★	★	★	★	★★	★	★	-	8
Jamil et al.	★	★	★	★	★★	★	★	-	8
Jeon et al.	★	★	★	★	★★	★	★	-	8
Jiménez Vacas et al.	-	-	-	★	★	★	★	-	4
Jung et al.	★	★	★	★	★★	★	★	-	8
Kang et al.	★	★	★	★	★★	★	★	-	8
Kim et al.	-	★	★	★	★	★	★	-	6
Kızılay et al.	-	-	★	★	★	★	★	-	5
Lin et al.	-	★	★	★	★★	★	★	-	7
Loeb et al.	★	★	★	★	★★	★	★	-	8
Lotan et al.	-	★	★	★	★	★	★	-	6

Luo et al.	-	-	★	★	★★	★	★	-	6
Malaeb et al.	-	★	★	★	★	★	★	-	6
May et al.	★	★	★	★	★★	★	★	-	8
Milanese et al.	-	-	★	★	★★	★	★	-	6
Mitsuzuka et al.	★	★	★	★	★★	★	★	-	8
Ohno et al.	-	★	★	★	★	★	★	-	6
Park et al.	★	★	★	★	★★	★	★	-	8
Rakic et al.	★	★	★	★	★★	★	★	-	8
Sato et al.	-	-	★	★	★	★	★	-	5
Shariat et al. (2004)	★	★	★	★	★★	★	★	-	8
Shariat et al. (2006)	-	-	★	★	★★	★	★	-	6
Shariat et al. (2007)	-	★	★	★	★★	★	★	-	7
Shin et al.	-	-	★	★	★	★	★	-	5
Van den Ouden et al.	★	★	★	★	★	★	★	-	7
Wang et al.	★	★	★	★	★★	★	★	-	8
Wang et al.*	-	★	★	★	★	★	★	-	6
Yamamoto et al.	★	★	★	★	★★	★	★	-	8
Yee et al.	★	★	★	★	★★	★	★	-	8

* Wang, F.; Liu, F.; Liang, J.; Yang, F.; Xing, N. Preoperative Platelet Count Correlates With Postoperative Perineural Invasion on Specimen in Patients Treated With Radical Prostatectomy. Front. Oncol. 2022, 12, 1–10, doi:10.3389/fonc.2022.906936.
